# Supplementary material for: Harnessing droplet microfluidics and morphology-based deep learning for the label-free study of polymicrobial-phage interactions
Source: Commun Biol. 2025 Nov 12;8:1556. doi: 10.1038/s42003-025-08925-9 (PMC12612114; doi:10.1038/s42003-025-08925-9)
Supplement: Supplementary file 2 — Supplementary Information [file 42003_2025_8925_MOESM2_ESM.pdf]

Supplementary Information

Harnessing droplet microfluidics and deep morphology-based learning for the label-free study of polymicrobial-phage interactions

Anuj Tiwari[1], An Mei Daniels[1,2], Remy Chait[1,2,3], Robyn Manley[3,*], Fabrice Gielen[1,4,*]

[1] Living Systems Institute, Faculty of Health and Life Sciences, University of Exeter, Exeter, UK

[2] Natural Sciences, Department of Physics and Astronomy, Faculty of Environment, Science and Economy, University of Exeter, Exeter, UK

[3] Department of Biosciences, Faculty of Health and Life Sciences, University of Exeter, Exeter, UK

[4] Department of Physics and Astronomy, Faculty of Environment, Science and Economy, University of Exeter, Exeter, UK

Contents

1. Comparison to microfluidics-based studies 2
2. Deep learning for cell morphology detection 3
   2.1 PA14 *ΔflgK* morphology detection model 4
   2.2 MSSA476 morphology detection model 5
   2.3 Polymicrobial model to detect both PA14 *ΔflgK* and MSSA476 using a single model 5
3. Removal of static detections 6
4. Time-lapse cell counts from individual droplets for PA-SA co-cultures 8
5. Bulk co-cultures of PA14 ΔflgK and MSSA476 9
6. One-step growth curve for phage P278 10
7. Time-lapse cell counts from individual droplets for bacteria-phage interactions 13
8. Interaction between MSSA476 and phage P278 done in a plate reader 14
9. Time-lapse cell counts from individual droplets for two species and phage P278 interactions 15
10. Deep learning for autofocus 16
11. Spheroplast-like detections during PA14 *ΔflgK* lysis 18

12. CFU/mL calibration for both species 20
13. Calculation of cell doubling times 23
14. Movies captions 24
References 25

1. Comparison to microfluidics-based studies

Recent innovations in microfluidics have enabled powerful approaches to study microbial communities. Within single phase devices, the mother machine devices have been extensively used. For instance, Alma Dal Co and Simon van Vliet have advanced the field with devices to study ecological interactions and feedback between environmental structure and microbial behaviour, though most systems rely on flow-through or 2D culturing formats. The Hallatschek's and Wacław's groups have focused on spatial dynamics and stochastic effects in bacterial colonies using planar microfluidic chambers and agarose-based devices, highlighting genetic drift and sectoring phenomena. Early droplet-based co-cultivation systems such as that of Herrera-Estrella *et al.* (2011) demonstrated the utility of fluorescently labelled bacteria for high-throughput mapping of microbial interactions in droplets, highlighting the potential of droplet platforms for studying microbial consortia.

There have also been innovations in droplet-based screening methods: the Hasty's lab has pioneered synthetic gene circuits and quorum sensing systems within droplet-based setups, often requiring fluorescent reporters.

In contrast, our approach uniquely combines long-term confinement of polymicrobial co-cultures in picolitre droplets with label-free time-lapse Z-stack imaging, automated deep-learning-based autofocus, and morphology-based species-specific detection using YOLOv5. Unlike fluorescence-reliant studies (e.g., co-encapsulated symbiotic bacteria), our method preserves native cell genetics and avoids labelling bias, enabling continuous quantification of growth and phage-induced lysis in mixed communities. This expands the applicability of microfluidic studies towards label-free infection dynamics in complex communities. A summary Table S1 listing the state-of-the-art microfluidics methods is given below.

Supplementary Table 1. Comparison of present study with relevant literature studying polymicrobial cultures using microfluidics methods.

Lab / Study	Microfluidic Format	Cell Detection Method	Polymicrobial Support	Phage Interaction Studied	Fluorescence Requirement	Quantitative Output
This Study	Anchored picolitre droplet arrays	Label-free deep learning (YOLOv5, morphology-based)	Yes (e.g., PA14 *ΔflgK* and MSSA476)	Yes (quantitative phage-bacteria dynamics in droplets)	No (fully label-free detection)	Growth curves, doubling time, lysis dynamics

Hallatschek (2019), [1]	Planar microfluidic chambers / agarose devices	Imaging / fluorescent reporters	Yes (coexistence, spatial segregation)	No or minimal (focus on population dynamics without phages)	Yes (often required for tracking)	Growth patterns, spatial segregation
Hasty *et al.* (2009), [2]	Droplets / microchambers with synthetic circuits	Fluorescent reporters (synthetic constructs)	Limited (focus on engineered *E. coli* strains)	Rarely (focus more on genetic circuits)	Yes (integrated with circuits)	Circuit dynamics, population trends
Ramachandran et al., 2024, [3]	Flow-based microchannels and trapping devices	Brightfield / fluorescent microscopy	Limited (mostly monocultures or passive transport studies)	No	Often yes	Transport, mixing, shear impact
Dal Co *et al.* (2019, 2020), [4,5]	Multilayer PDMS devices for ecological studies	Brightfield, phase contrast, fluorescent	Yes (spatially structured communities)	No	Yes (common)	Spatial structure, ecological stability
Park *et al.* (2011) [6]	Droplet-based co-cultivation in microwell array	Fluorescent imaging	Yes (symbiotic interactions in co-encapsulated communities)	No	Yes (fluorescent reporters used)	Co-culture viability and interaction network mapping

2. Deep learning for cell morphology detection

For YOLOv5, precision and recall are calculated using standard definitions from the field of object detection. Precision is the fraction of true positive detections out of all positive detections made by the model. Mathematically, it can be expressed as:

Precision = True Positive / (True Positive + False Positive)

where True Positive is the number of correctly predicted objects, and False Positive is the number of objects predicted by the model that do not exist in the ground truth data.

Recall is the fraction of true positive detections out of all ground truth positives. Mathematically, it can be expressed as:

Recall = True Positive / (True Positive + False Negative)

where True Positive is the number of correctly predicted objects, and False Negative is the number of objects in the ground truth data that were not detected by the model. Other important measures for measuring the performance of a trained model are training loss and validation

loss. IOU is defined as the ratio of the intersection area between the predicted bounding box and the ground truth bounding box to the union area of these two boxes. It is expressed mathematically as:

IOU = (Area of intersection) / (Area of union)

Mean average precision (mAP) values are measured as a function of IOU values. mAP_50 refers to the precision of detection at 50% IOU threshold.

While the general trend for a well-trained object detection model for precision and recall is an increase close to 1 over the training epochs, the trend for training and validation loss is a decrease towards 0. For each model, the training conditions are mentioned in each section below.

2.1 PA14 *ΔflgK* morphology detection model

The dataset was generated by taking images from PA14 *ΔflgK* growth and lysis experiments. A total of 230 images containing over 1600 examples of PA14 *ΔflgK* morphology in different microscopic conditions (light intensity, gain, collimator position) were labelled manually by drawing bounding boxes. This dataset was then split into a ratio of 70:30 for training and validation respectively. For PA14 *ΔflgK* model, even without the transfer learning, we reached a mAP_50 value of 99.5%. The total precision and recall values at the end of the training were 0.99 and 0.98 respectively. The final values of training and validation loss were 0.0146 and 0.008 demonstrating effective training. The training metrics are shown in Supplementary Figure 1.

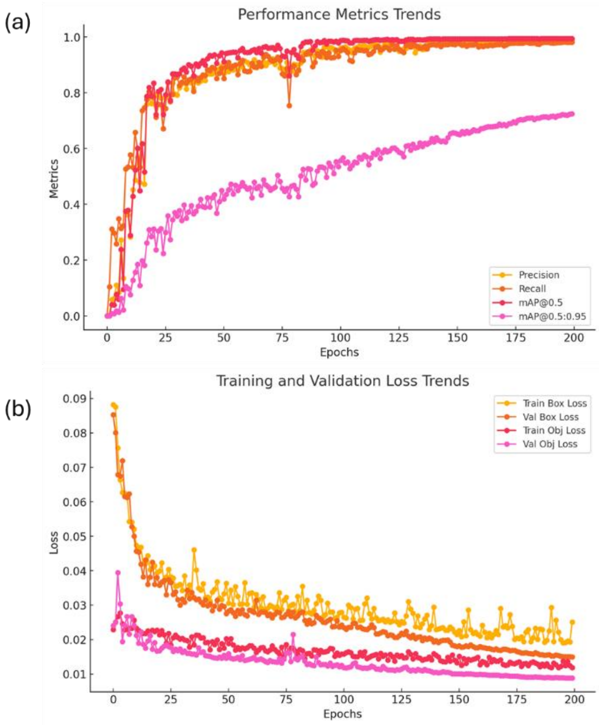

Supplementary Figure 1: Training metrics for PA14 *ΔflgK* morphology detection model

2.2 MSSA476 morphology detection model

A total of 150 images containing over 1300 examples of MSSA476 morphology in different microscopic conditions were labelled and split into a 70:30 ratio like the PA14 *ΔflgK* model. The training was performed in two iterations using transfer learning. The original model was trained for over 200 epochs after freezing the first 10 layers of the model. The mAP_50 obtained at the end of this step was 83.6% with training and validation loss at 0.025 and 0.019 respectively as seen in Supplementary Figure 2(a) and 2(b). Once the training finished, the best weights were obtained from the first training and retrained for 60 epochs after unfreezing the first 10 layers to improve mAP values. After the final training with the transfer learning step, the final mAP_50 was 90.5% with the training and validation loss values at 0.023 and 0.017 as seen in Supplementary Figure 2(c) and 2(d). This model was used for detection of MSSA476 cocci-shaped morphology.

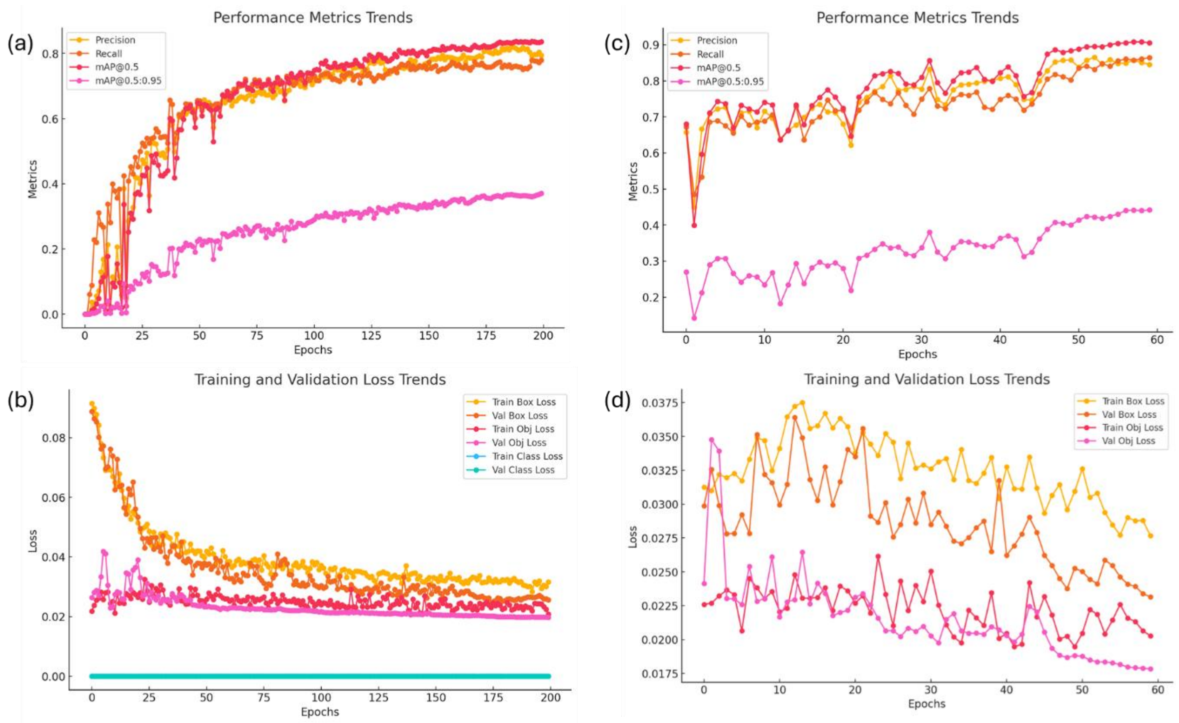

Supplementary Figure 2: Training metrics for MSSA476 morphology detection. (a-b) Training metrics for the first 200 epochs where the first 10 layers of the model were frozen (c-d) Training metrics after obtaining weights from the first training iteration and retraining them after unfreezing the layers of the model.

2.3 Polymicrobial model to detect both PA14 *ΔflgK* and MSSA476 using a single model

The third model was trained for detection of both PA14 *ΔflgK* and MSSA476 cell strains. The dataset consisted of 480 images with over 2000 examples of PA14 *ΔflgK* morphology and 2200 examples of MSSA47 morphology. This dataset was generated by combining the first two

datasets as well as adding examples from polymicrobial experiments where both PA14 *ΔflgK* and MSSA476 cells were present in one droplet. Similar to the MSSA476 model, this model was also trained using transfer learning. The first iteration of training was done for 150 epochs after freezing the first 10 layers of the model. The mAP value obtained was 91.5% with a training and validation loss of 0.016 and 0.001 as seen in Supplementary Figure 3(a) and 3(b). The transfer learning involved training for another 60 epochs after unfreezing the first 10 layers. This improved the mAP value to 97% with a training and validation loss of 0.013 and 0.0005 as seen in Supplementary Figure 3(c) and 3(d).

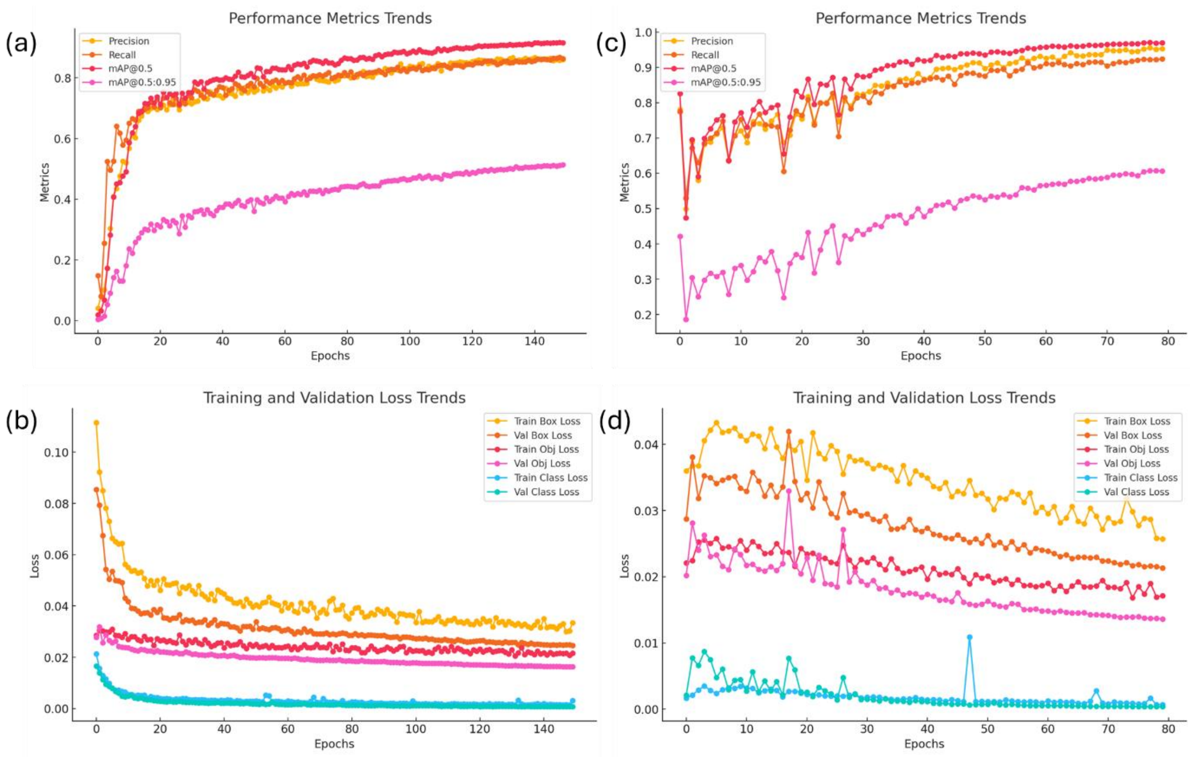

Supplementary Figure 3: Polymicrobial model training with transfer learning. (a-b) Training metrics for the first 150 epochs where the first 10 layers of the model were frozen (c-d) Training metrics after obtaining weights from the first training iteration and retraining them after unfreezing the layers of the model

3. Removal of static detections

We used a Density-Based Spatial Clustering of Applications with Noise (DBSCAN) algorithm to cluster detections that were spatially stable across time series so these incorrect detections could be excluded from the analysis. We set a threshold of 20% of detections over the entire time series found at the same location to classify a detection as static. They likely corresponded to false detections as cells are expected to be slightly motile and/or undergo Brownian motion. These detections were removed from the final cell counts.

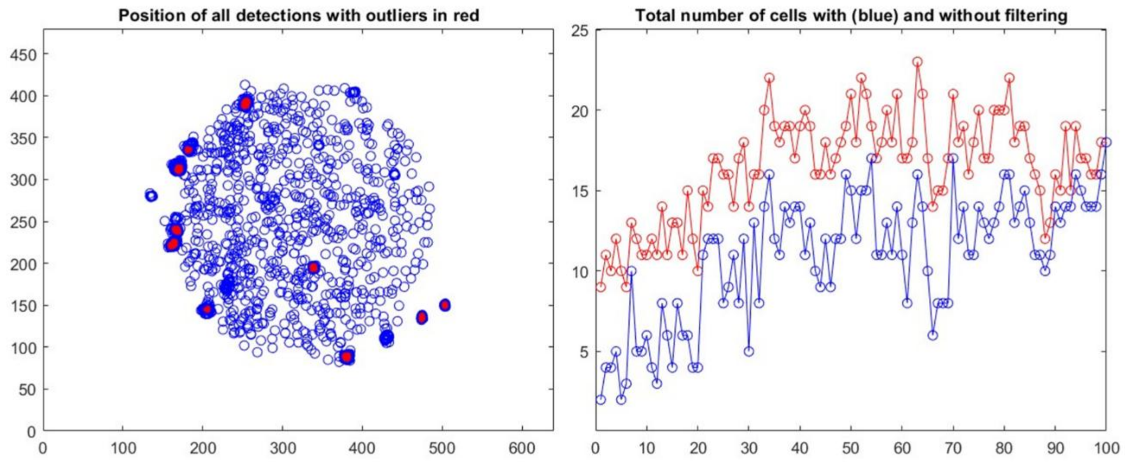

Supplementary Figure 4: Removal of static and recurring false detections **A.** Detections remaining close to the same location are detected with a clustering algorithm. **B.** Example difference in counts with and without the static detection removal.

4. Time-lapse cell counts from individual droplets for PA-SA co-cultures

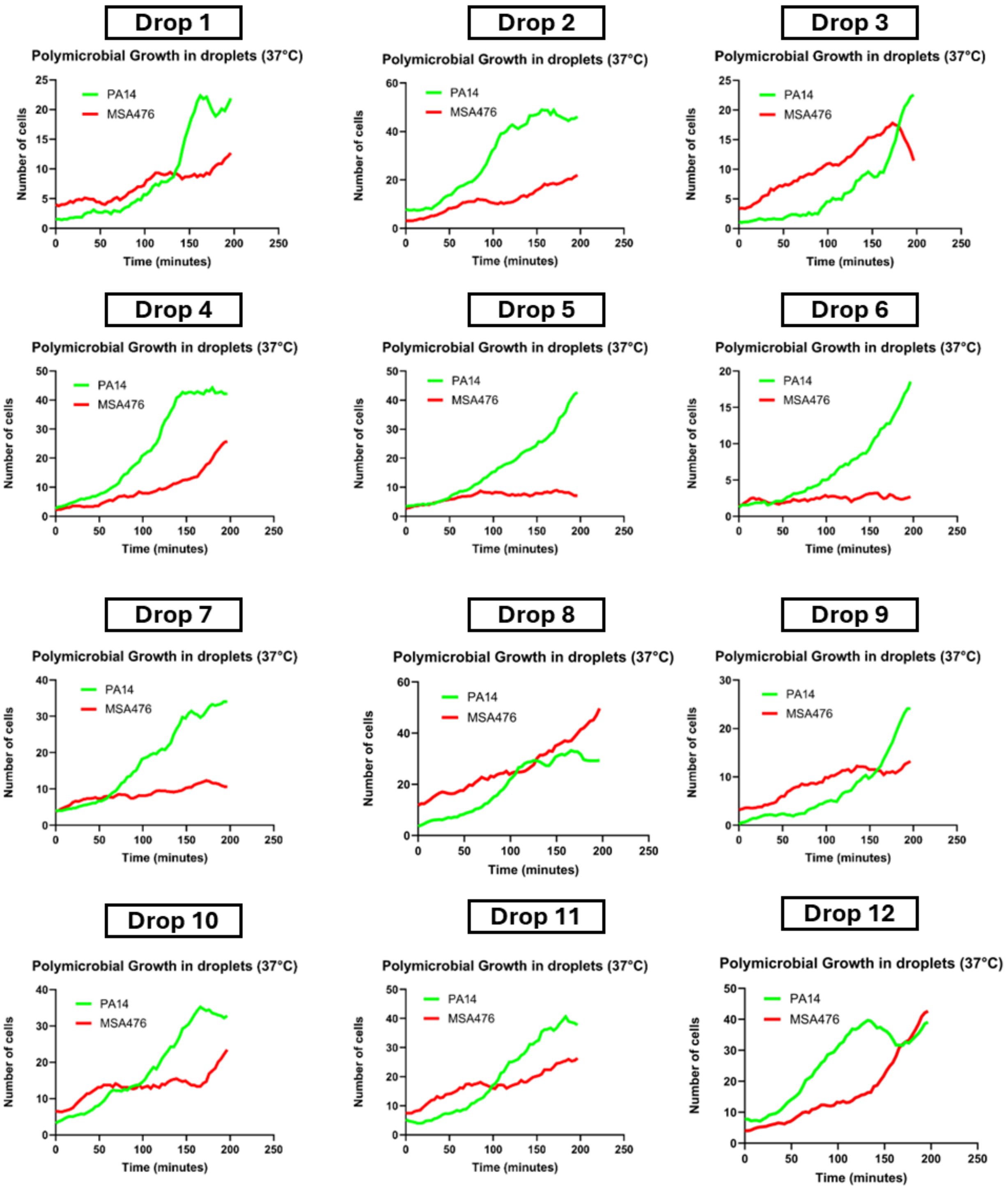

Supplementary Figure 5: PA-SA co-culture growing in LB droplets showing the moving average of individual number of PA14 *ΔflgK* and MSSA cells observed over time in 12 droplets. *S. aureus* outnumbered *P. aeruginosa* at end point for droplets 8 and 12.

5. Bulk co-cultures of PA14 ΔflgK and MSSA476

We have performed bulk co-culture experiments of *P. aeruginosa* PA14 *ΔflgK* and *S. aureus* MSSA476 in the same LB medium and incubation conditions in microtiter plates. These data (Supplementary Figure 6) show that while PA14 *ΔflgK* grows comparably in both mono- and co-cultures, MSSA476 reaches significantly lower densities in polycultures, with its population reduced by over an order of magnitude after 24 hours. This outcome aligns with previous literature reporting *S. aureus* suppression by *P. aeruginosa* in bulk formats [7].

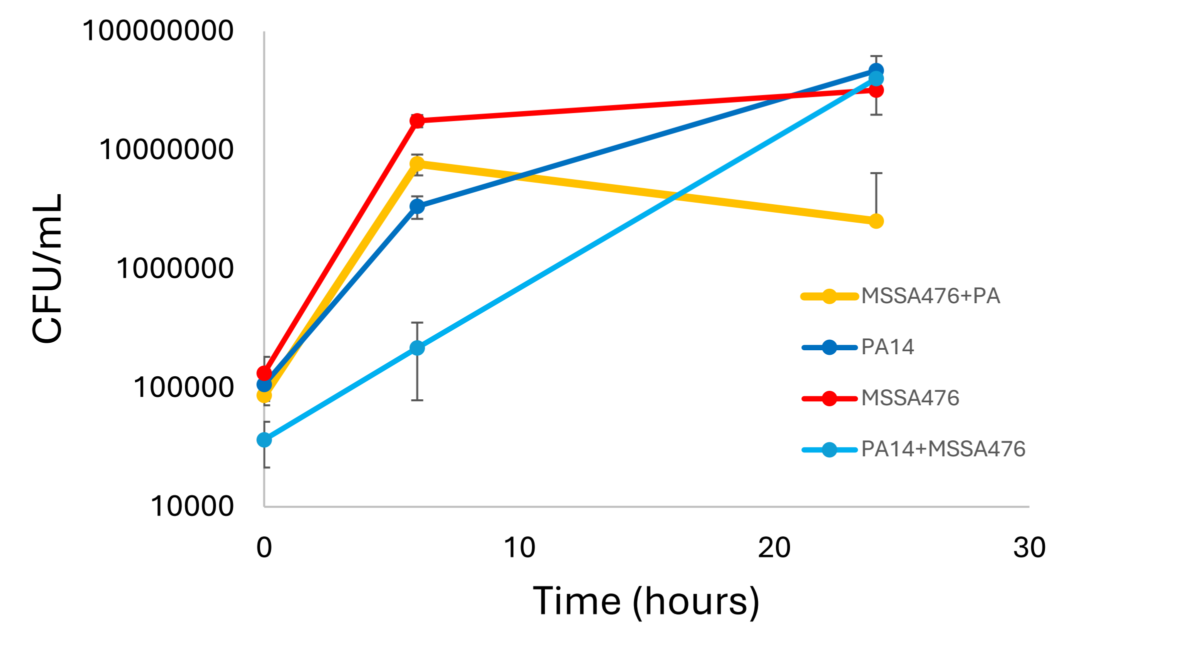

Supplementary Figure 6. Evolution of CFU/mL for PA14 *ΔflgK* and MSSA476 growing either in monoculture or in presence of each other. 3 technical replicates were performed per time point.

6. One-step growth curve for phage P278

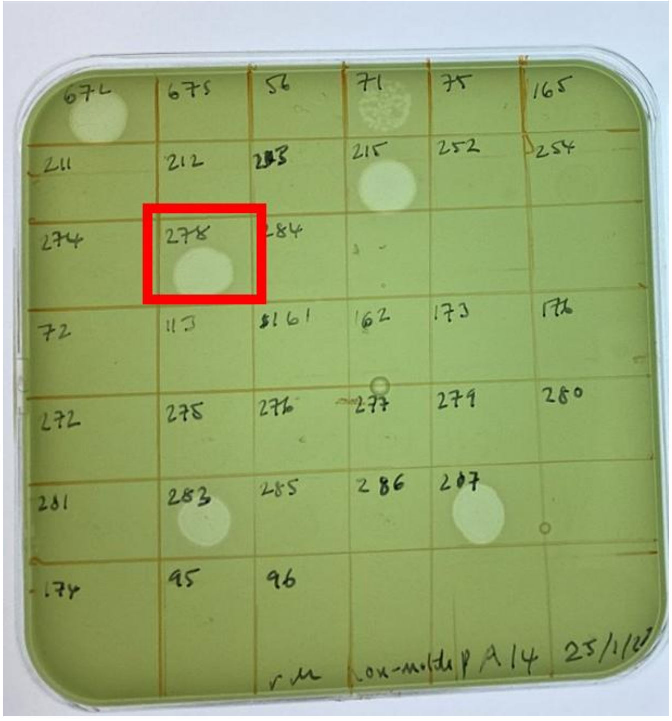

Supplementary Figure 7: Characterizing 35 phages from the Exeter Citizen Phage library against PA14 *ΔflgK* strain using the spot assay [8]. Clear spots are indicative of PA14 *ΔflgK* lysis.

P278 was chosen as it formed clear plaques on the top agar bed with PA14 *ΔflgK* cells.

To characterize phage P278 for time to lysis and burst size, we performed a standard one-step growth curve assay. A single colony of PA14 *ΔflgK* cells was grown in 5 mL of LB media overnight. 50 µL of cells from overnight culture were diluted into 5 mL of fresh LB media. The cells were grown up to an OD_{600} of 0.650 corresponding to 6 x 10^8 CFU/mL. The phage titer for P278 had a concentration of 8 x 10^9 PFU/mL. 24 Eppendorf tubes with volumes 1.5 mL were autoclaved and prepared for the experiment. Each Eppendorf tube would contain 5 µL of phage titer, 332 µL of cells at OD 0.65 and 663 µL of fresh LB bringing the volume to a total of 1 mL. This would result in an MOI of 0.2 in each tube. All 24 tubes were placed on a heating block set at 37 °C, shaking at 200 RPM. We chose to sample 8 points over the duration of 1 hour; each point measured every 8 minutes with 3 repeats. For each point, 200 µL of solution was pipetted into a 96 well filter plate and centrifuged at 1200 g for 2 minutes to separate out the free phage left in the solution. After repeating this process across 8 time-points over 56 minutes, a 10-fold dilution series in SM media was performed for each point 8 times.

LB agar-based Petri dishes were prepared to perform the plaque forming assay to calculate the number of free phages for each time point. 100 µL of PA14 *ΔflgK* cells at OD 0.7 were mixed with 5 µL of $CaCl_2$ and $MgCl_2$ and 5 mL of soft agar and poured on top of each petri dishes with LB agar base to create a bed of PA14 *ΔflgK* cells as a top layer. Using a multichannel pipette, 2.5 µL of each concentration from the dilution series for each point was plated onto the

Petri dishes and left overnight to calculate the concentration of free phage as seen in Supplementary Figure 8. The resulting number of plaque-forming units (pfu) are listed in Table S3.

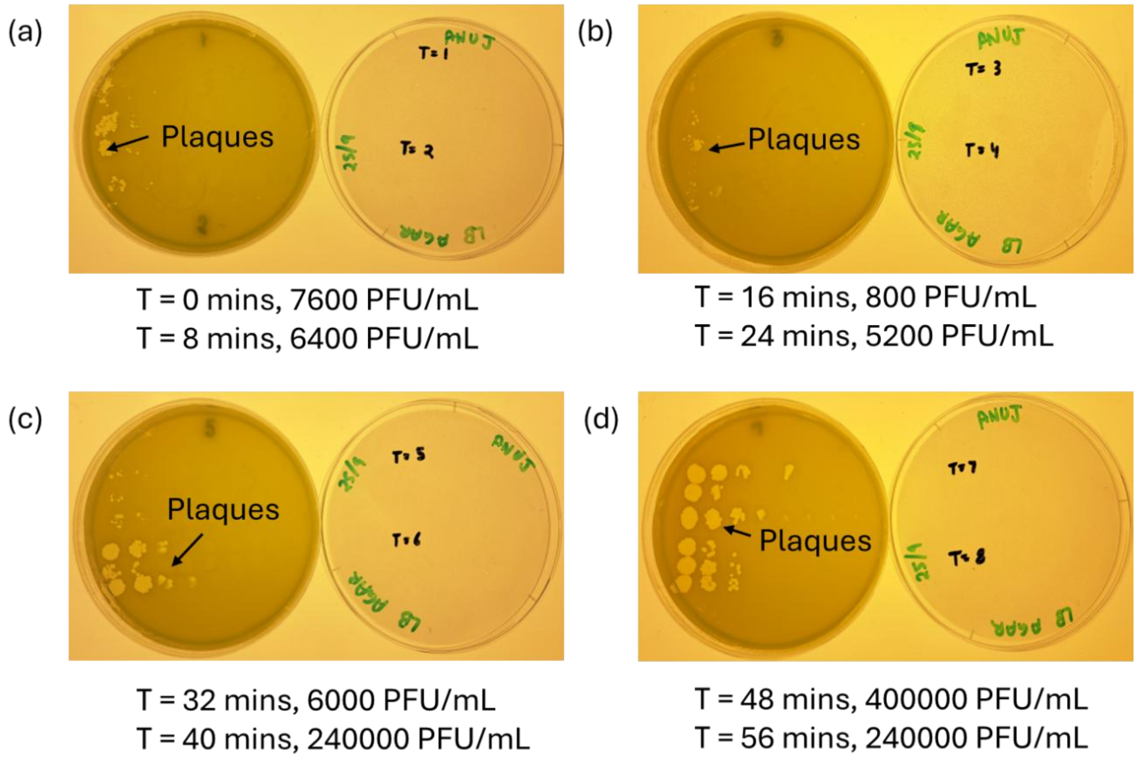

Supplementary Figure 8: One-step growth curve assay to characterize P278 phage and its interaction with PA14 *ΔflgK* cells. (a) Time points 1 and 2 with dilution series and plaques formed. (b) Time points 3 and 4 with dilution series and plaques formed (c) Time points 5 and 6 with dilution series and plaques formed. (d) Time points 7 and 8 with dilution series and plaques formed.

Supplementary Table 2: PFU count overtime points

**Time (mins)**	**PFU**
0	7600
8	6400
16	8000
24	5200
32	6000
40	240000
48	400000
56	240000

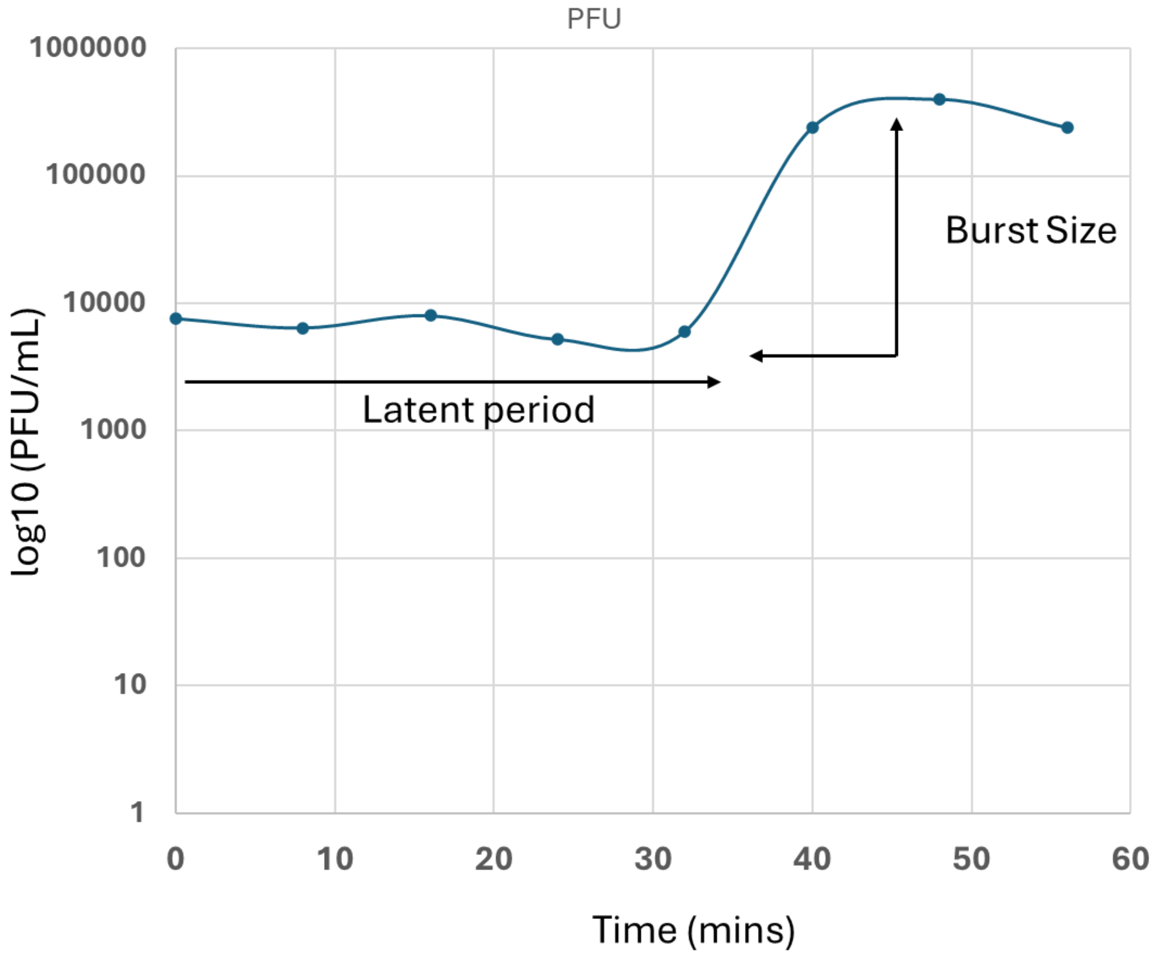

Supplementary Figure 9. Extraction of burst size from the P278 one-step growth curve calculated by dividing the average pfu/mL after the rise period by the average pfu/mL during the latent phase.

Taking the PFU values listed in Table S3, we get the calculation as:

P278 burst size $= \frac{PFU_{end}}{PFU_{initial}} = \frac{3 \ x \ 10^5}{6.6 \ x \ 10^3} = 45.45 \sim 45$

The burst size for phage P278 was calculated at 45 and the time to first burst was observed to be ~37 mins as seen in Supplementary Figure 9.

7. Time-lapse cell counts from individual droplets for bacteria-phage interactions

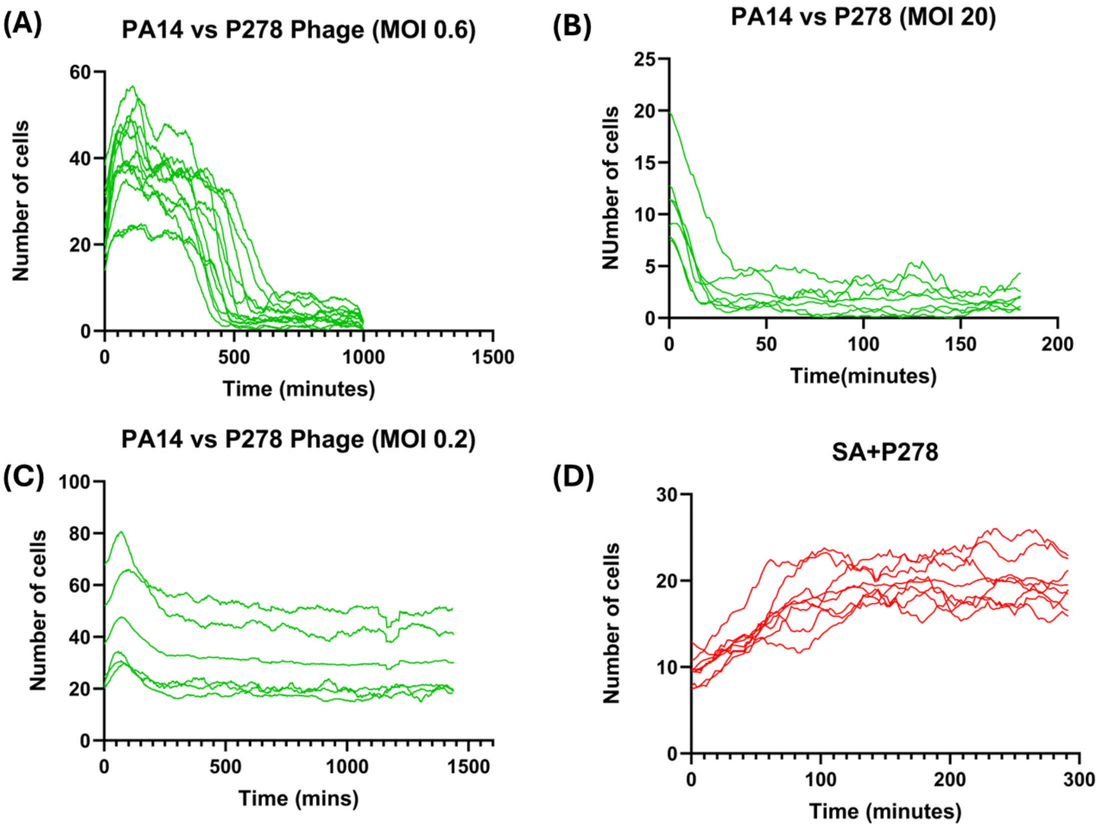

Supplementary Figure 10: (A)-(C) Interaction of PA with P278 at different phage concentrations with varying cell numbers in droplet (D) Interaction of SA with phage P278 equivalent to an MOI of 0.2.

8. Interaction between MSSA476 and phage P278 done in a plate reader

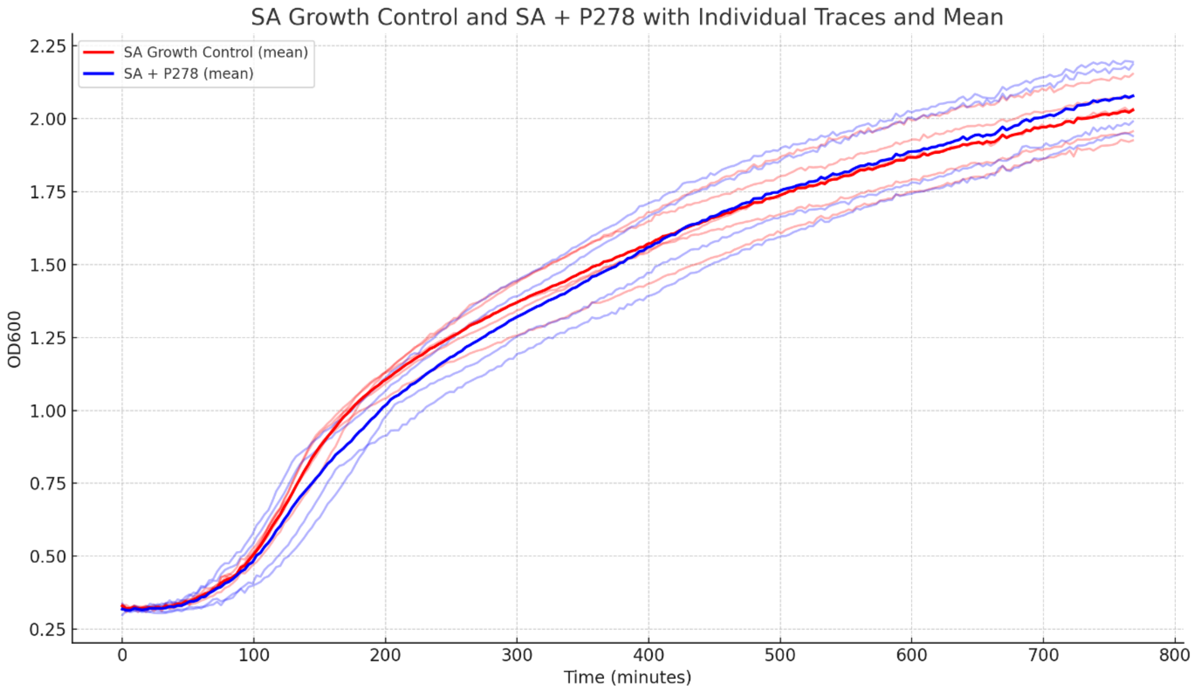

Supplementary Figure 11: Plate reader assay for interaction between MSSA476 cells and P278 phage

9. Time-lapse cell counts from individual droplets for two species and phage P278 interactions

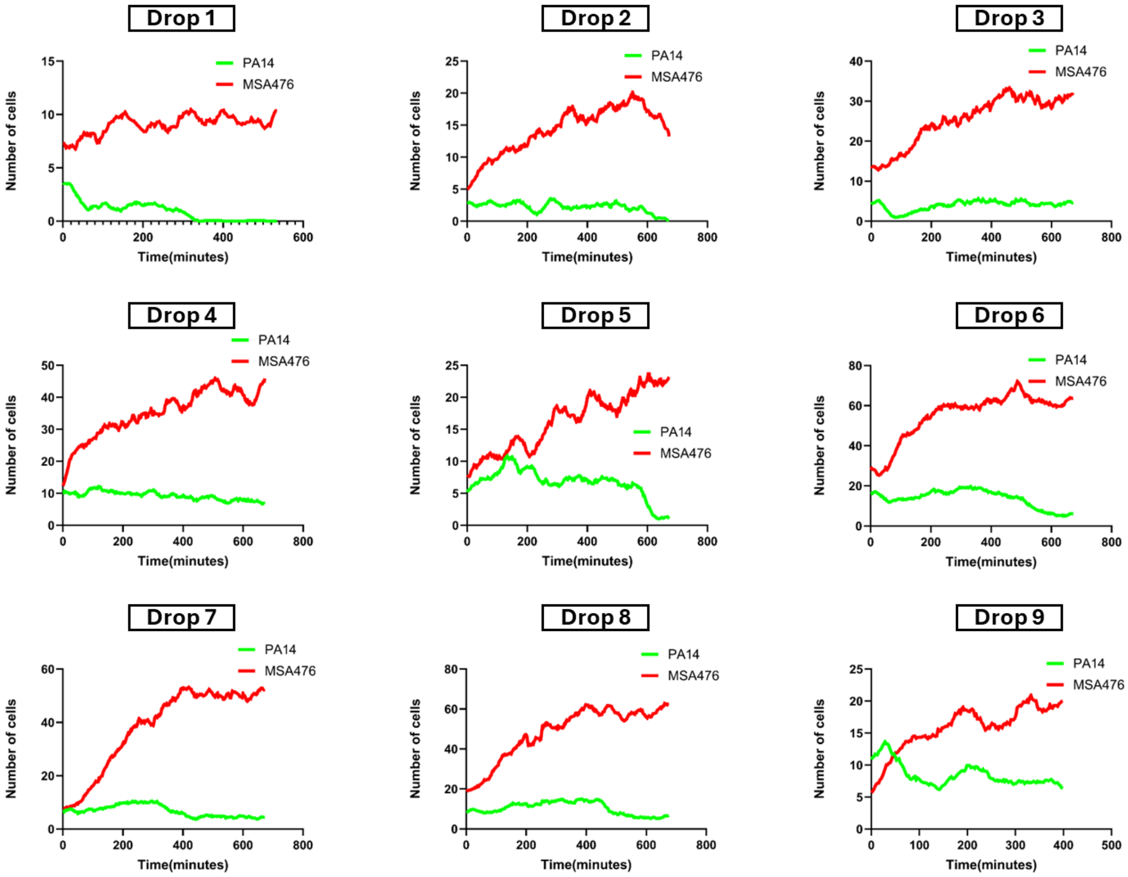

Supplementary Figure 12: PA-SA co-culture with P278 phage in LB droplets showing the moving average of individual number of PA14 *ΔflgK* and MSSA cells observed over time in 9 droplets at MOI 2.5

s

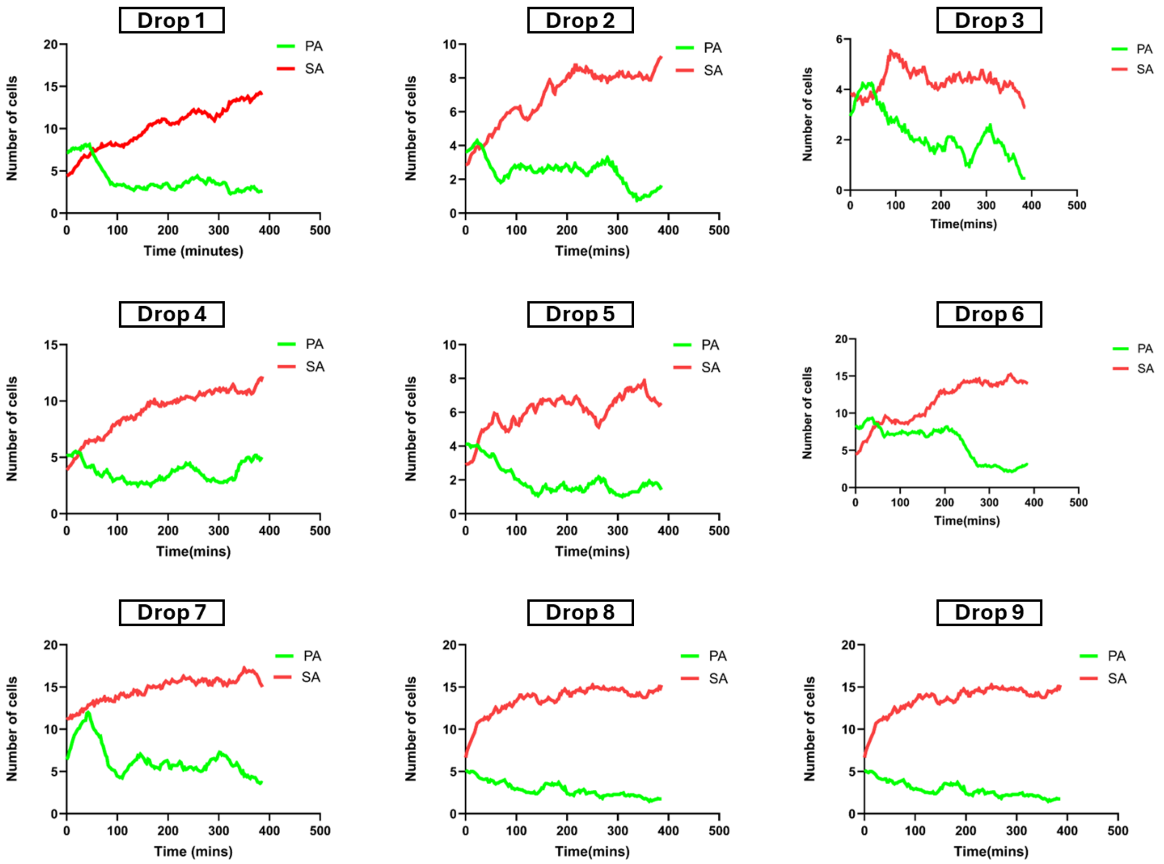

Supplementary Figure 13: PA-SA co-culture with P278 phage in LB droplets showing the moving average of individual number of PA14 *ΔflgK* and MSSA cells observed over time in 9 droplets at MOI 5.

10. Deep learning for autofocus

An initial training of the image classification model using YOLOv8-Nano for autofocus u was used with a set of 300 images trained for 160 epochs. 5 successive training iterations of 55 epochs each were then carried out, with additional training images from model failures added each time, making the model more robust in its final iteration.

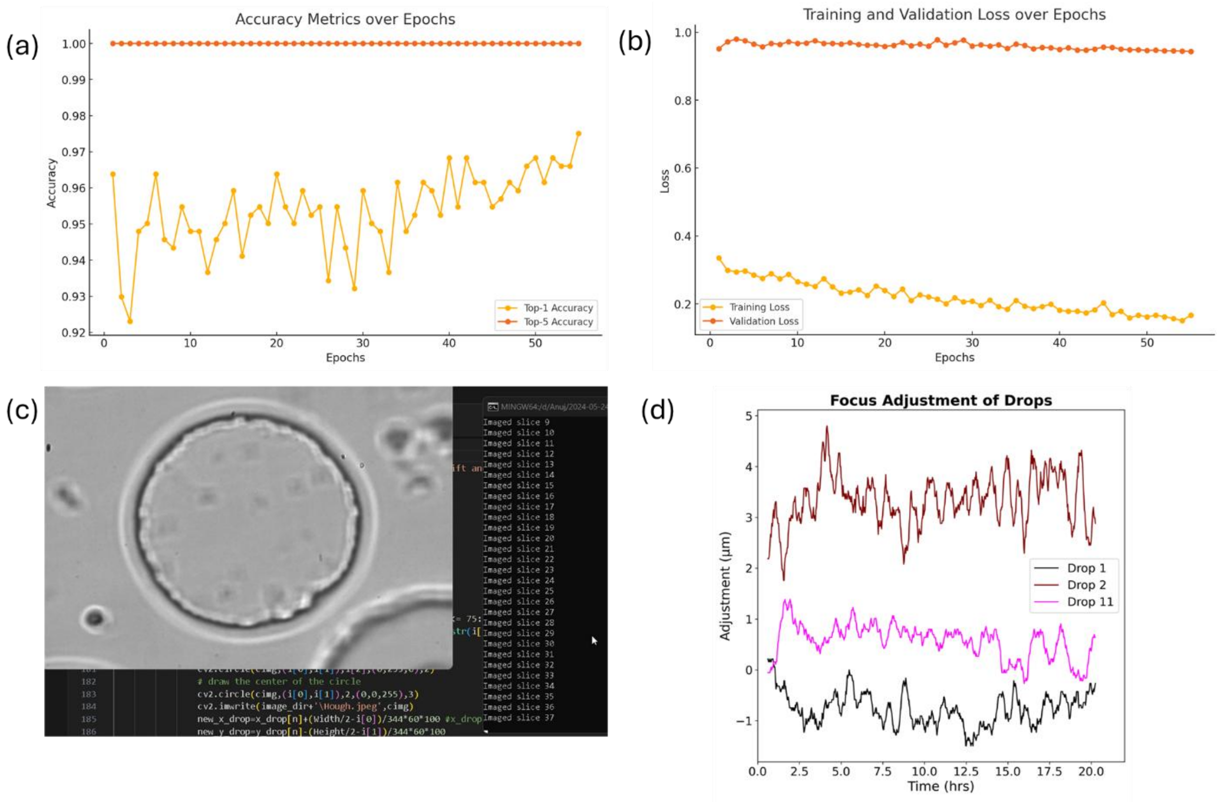

Supplementary Figure 14: (a) Accuracy metrics over the last 55 epochs of training for image classification models for autofocus (b) Training and validation loss over the last 55 epochs for image classification training for autofocus model (c) Example of focus autocorrection during the experiment (d) Example of Z axis adjustments per droplet to correct focal drift during data acquisition experiments over 20 hours

Top 1 classification accuracy is the measure of the percentage of times the model's single highest-probability prediction (the "top 1" prediction) matches the true label. It is calculated as:

$$Top\ 1\ accuracy = \frac{Number\ of\ correct\ top{-}1\ prediction}{total\ number\ of\ samples} \qquad \text{(Eq. 1)}$$

In our case, the image classification model using YOLOv8-Nano reached a top 1 accuracy of **97.51%** over the training as seen in Supplementary Figure 14(a). Since there were only 5 classification classes, the top 5 accuracy remained 100% over the 50 epochs as seen in Supplementary Figure 14(a). The final training loss was observed to be 0.16 and final validation loss reached a value of 0.94 indicating strong model performance with robust learning and accurate predictions.

11. Spheroplast-like detections during PA14 *ΔflgK* lysis

To demonstrate further use of the morphology-based detection method, we have focused on the transition of PA14 *ΔflgK* from rod-shape to round cell morphology during the phage lysis process. We hypothesise that these round 'cells' are spheroplasts because of their characteristic round shape and lower contrast of their membrane following digestion of the peptidoglycan membrane by the phage endolysins (Supplementary Figure 15). However, further confirmation would be required to confirm spheroplasts such as membrane staining or extraction followed by peptidoglycan quantification. We found that these round cells were not detected in all experiments, indicating that there may be sometimes short lived. In the experiments where we could detect them, the number of spheroplasts-like cells in a droplet approximately matched the number of cells being lysed, indicating that the round morphology is a reproducible intermediate shape during the overall lysis process.

Significantly, phage particles may remain encapsulated within these spheroplast-like cells, delaying the release of newly synthesized phages and therefore delaying the lysis of other cells. This observation may help in deciphering lysis dynamics.

To quantify the detection of these round cells, we labelled 250 round-shape PA14 *ΔflgK* cells across 50 images and trained a separate YOLOv5 model. The mAP was 96% at IoU 0.5 with 50 epochs. An example lysis experiment at MOI 20 in which round shape cells are seen is displayed in Supplementary Figure 15 below (c.f. Table 2 in main manuscript, 'PA14 *ΔflgK* lysis 1'). We analysed 3 droplets at MOI 1 and 3 droplets at MOI 20 and found similar trends (Supplementary Figure 15 B).

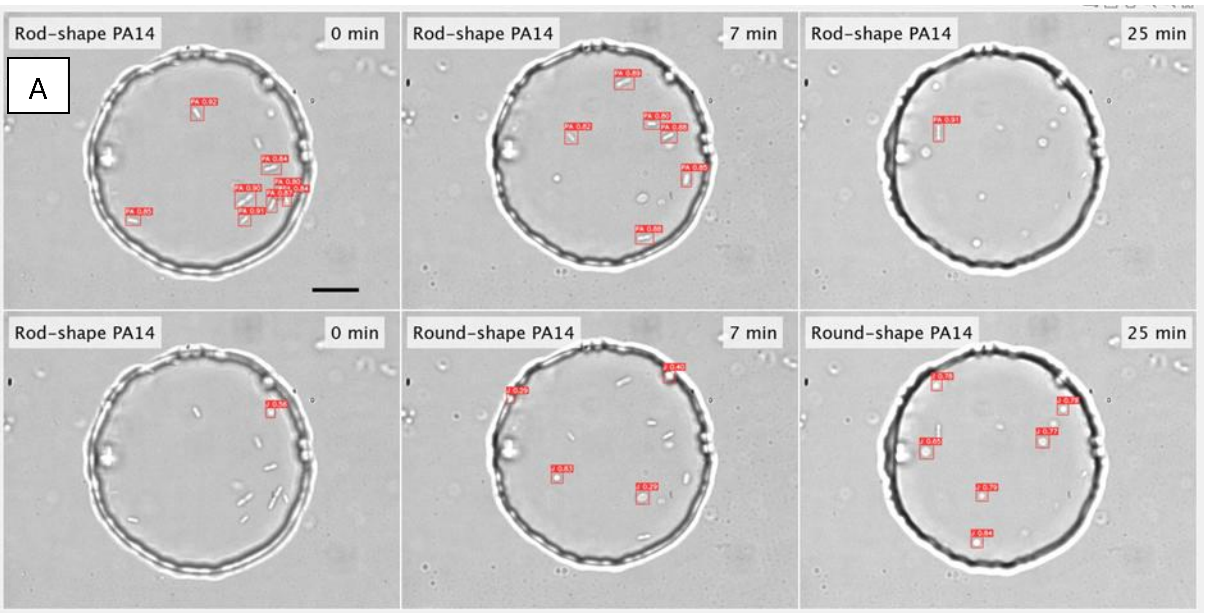

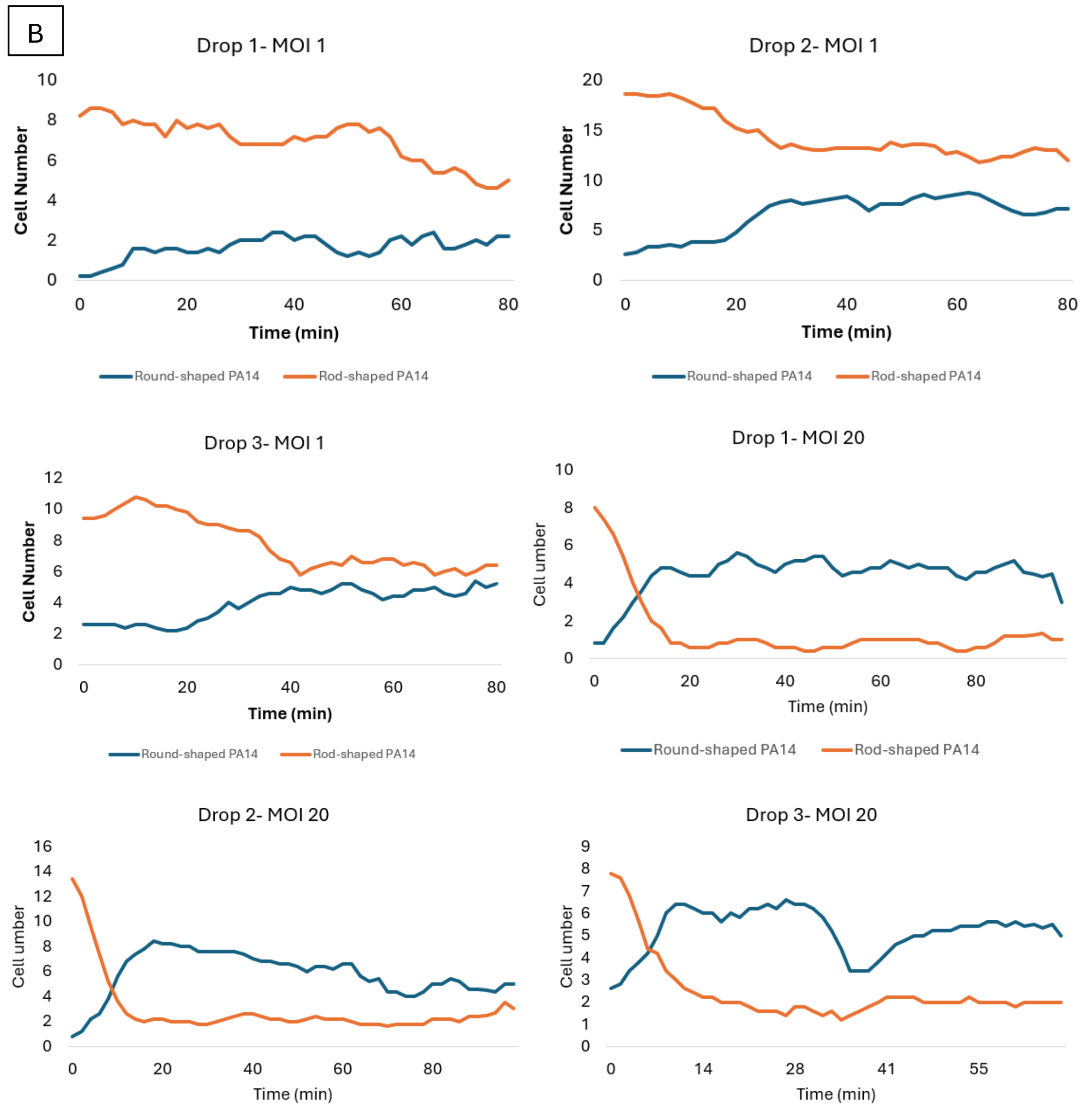

Supplementary Figure 15. A. Example time-lapse images of PA14 *ΔflgK* being lysed by P278. Round-shaped cells are detected with a separate YOLOv5 model. Scale bar:10 microns. **B.** Moving average over 5 time points for the number of detections of both round and rod-shaped PA14 *ΔflgK* cells during lysis experiments for MOI 1 and MOI 20.

However, when trying to detect spheroplasts-like PA14 *ΔflgK* cells in a co-culture of PA14 *ΔflgK* and MSSA, we found that the model could not accurately distinguish round cells from MSS476 due to their round morphologies being too similar.

12. CFU/mL calibration for both species

We have performed calibration for colony forming units per ml versus optical density measurements using our Clariostar plate reader for both species. We have pooled all the OD_{600} versus CFU/mL measurements in a single graph to get an accurate linear fit. This is shown in the Supplementary Figure 16 below. We note that there is a roughly linear correlation between CFU/mL and OD_{600} for the OD_{600} range we tested. All the data presented in Table S1 were obtained the day of the experiments by plating appropriate cell dilutions.

Supplementary Table 3. Optical density versus CFU/mL for PA14 *ΔflgK*

OD_{600}	CFU/mL (PA14 *ΔflgK*)
0.1	0.045E+08
0.15	0.6E+08
0.2	1.15E+08
0.25	1.71E+08
0.3	2.26E+08
0.35	2.81E+08
0.4	3.37E+08
0.45	3.92E+08
0.5	4.47E+08
0.55	5.02E+08
0.6	5.58E+08
0.65	6.13E+08
0.7	6.68E+08
0.75	7.24E+08
0.8	7.79E+08
0.85	8.34E+08
0.9	8.9E+08
0.95	9.45E+08
1	1E+09

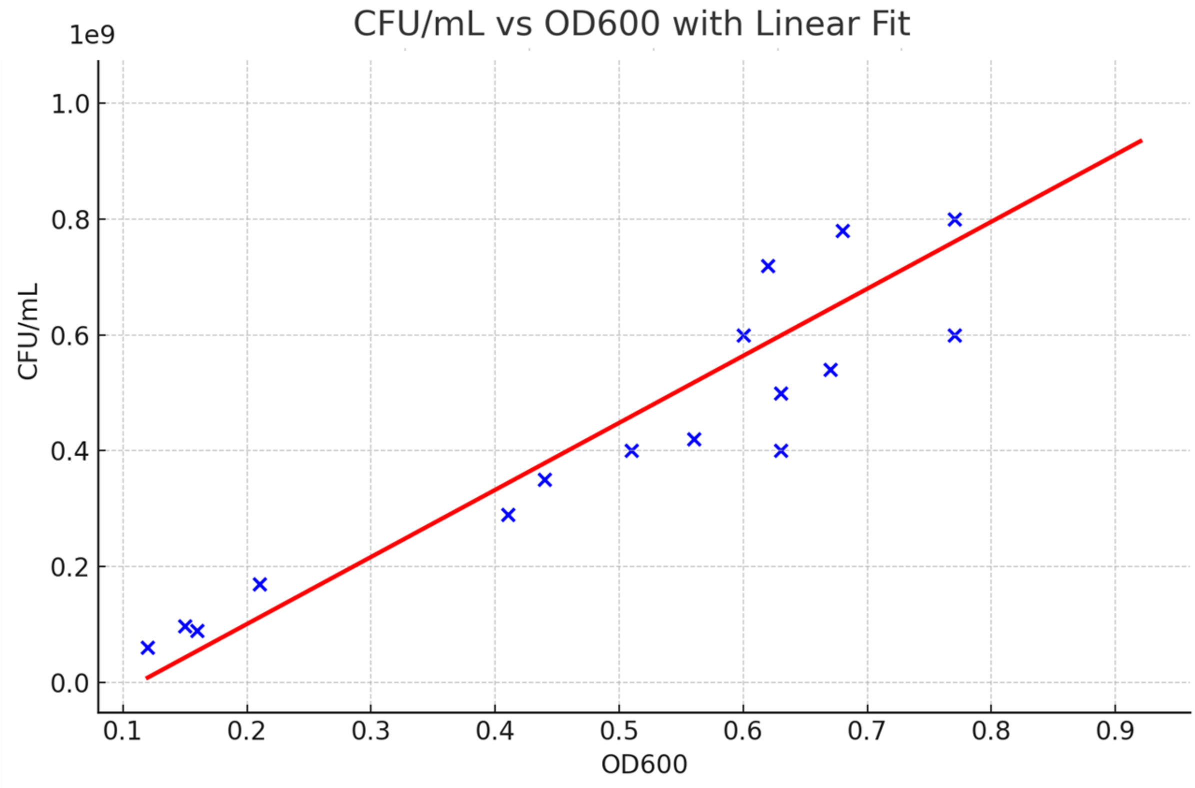

Supplementary Figure 16. Calibration OD600 versus CFU/mL for PA14 *ΔflgK* based on Table S1

The equation for the linear fit we obtained was **$CFU/mL = 1.16\times10^9 \times OD_{600} - 1.31\times10^8$** with an **$R^2$** value of **0.98**. Therefore, we used this conversion for PA14 to predict the average number of cells per droplet. We note that this correlation matches literature values for a closely related strain [9].

Supplementary Table 4. Optical density versus CFU/mL for MSSA476

OD_{600}	CFU/mL (MSSA476)
0.13	1.10E+07
0.14	1.30E+07
0.16	1.50E+07
0.17	1.80E+07
0.2	2.50E+07
0.35	4.90E+07
0.4	5.70E+07
0.67	9.00E+07
0.69	1.20E+08
0.85	1.30E+08
1.14	1.80E+08

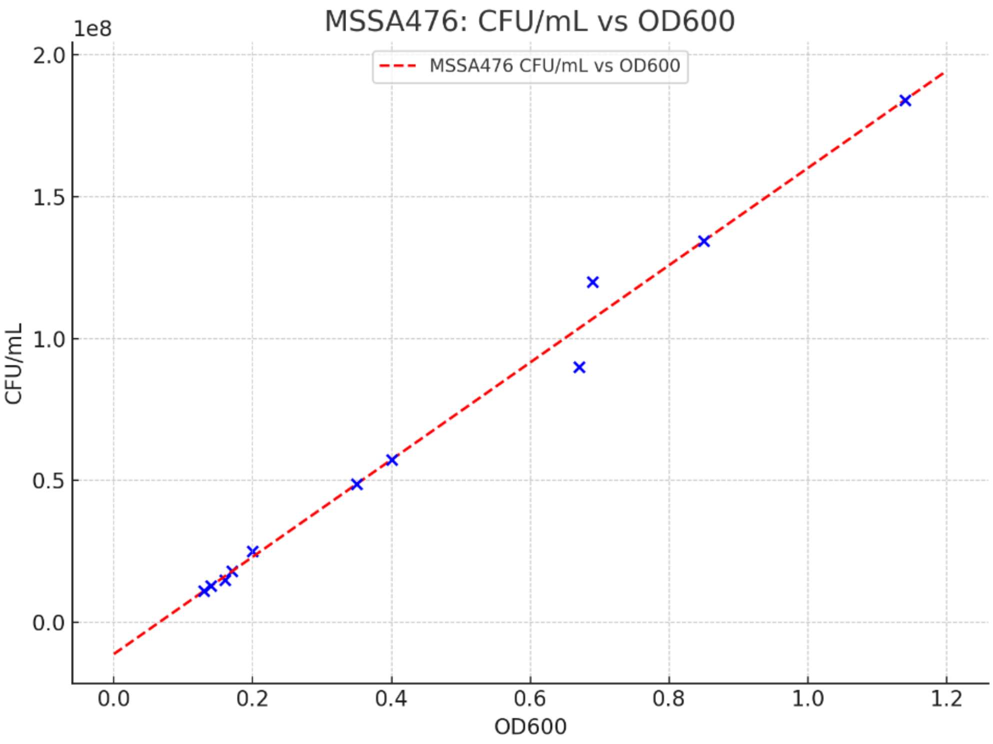

Supplementary Figure 17. Calibration OD_{600} versus CFU/mL for MSSA476 based on Table S2.

We have performed a similar analysis with MSSA476 and found the following relationship: **CFU/mL = 1.71 × 10⁸ × OD₆₀₀ − 1.11 × 10⁷** with an R^2 value of **0.96**. Table S2 has the corresponding CFU/mL values as separate entries.

13. Calculation of cell doubling times

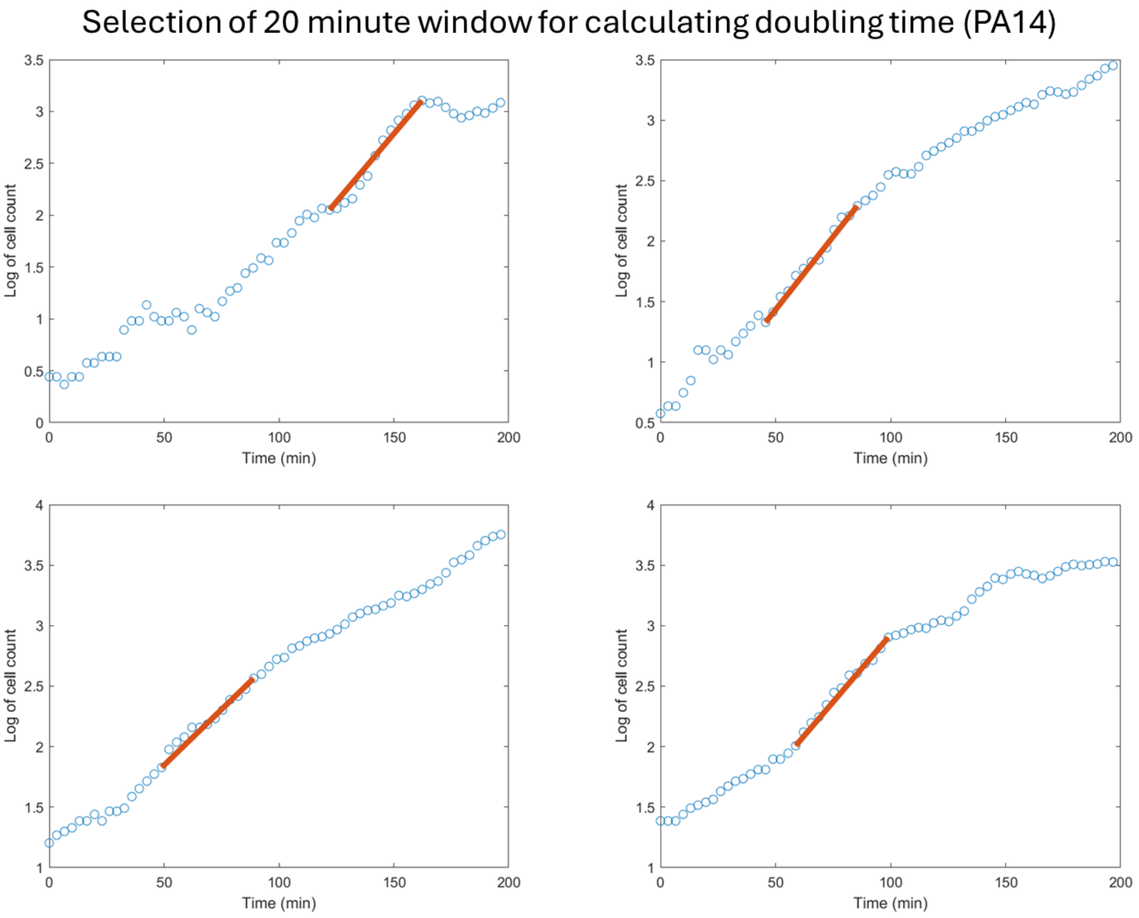

Supplementary Figure 18. Examples of maximum slope detection indicated by a red line corresponding to a 20 minute window for PA14 *ΔflgK* cell strain.

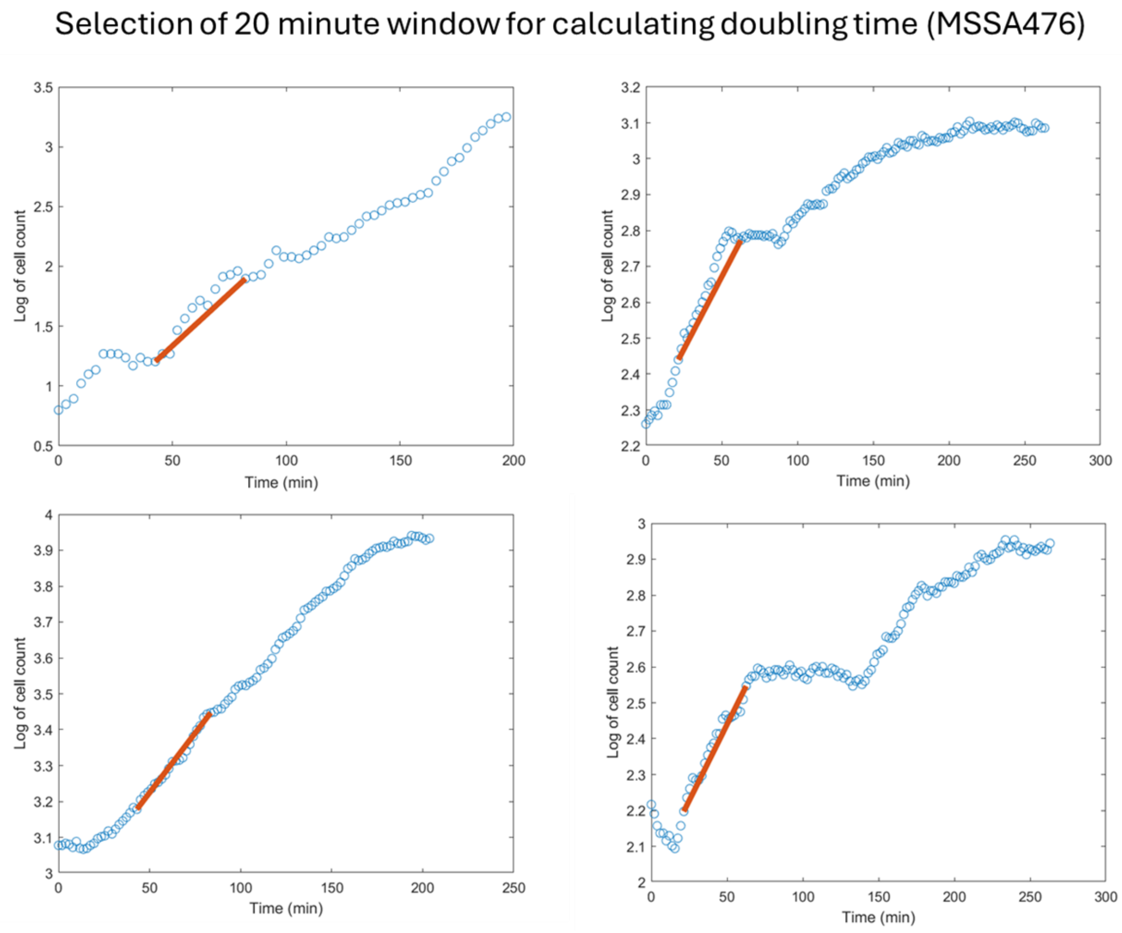

Supplementary Figure 19. Examples of maximum slope detection indicated by a red line corresponding to a 20 minute window for MSSA476 cell strain.

Figure S19 shows examples of selection of maximum slope (maximum specific rates). The MATLAB script (Growth_rate.m) is included on the project GitHub page.

14. Supplementary Movies captions

Movie S1. Example focus correction being applied before Z-stack imaging of every droplet with output of the python software displayed.

Movie S2. Time-lapse brightfield images illustrating the growth of a co-culture within a droplet at different time points, with *P. aeruginosa* cells marked by green dots and *S. aureus* cells by red dots. Corresponding cell counts are plotted on the right-hand panel.

Movie S3. Lysis of PA14 Δ*flgK* by P278 phage in droplet at MOI 0.6. Corresponding cell counts are plotted on the right-hand panel.

Movie S4. Effect of P278 phage on polymicrobial populations of PA14 Δ*flgK* and MSSA476 at MOI 2.5. Corresponding cell counts are plotted on the right-hand panel.

Supplementary References

1 Gralka, M. & Hallatschek, O. Environmental heterogeneity can tip the population genetics of range expansions. *Elife* **8**, doi:10.7554/eLife.44359 (2019).

2 Bennett, M. R. & Hasty, J. Microfluidic devices for measuring gene network dynamics in single cells. *Nat Rev Genet* **10**, 628-638, doi:10.1038/nrg2625 (2009).

3 Ramachandran, A., Stone, H. A. & Gitai, Z. Free-swimming bacteria transcriptionally respond to shear flow. *Proc Natl Acad Sci U S A* **121**, e2406688121, doi:10.1073/pnas.2406688121 (2024).

4 Dal Co, A., van Vliet, S., Kiviet, D. J., Schlegel, S. & Ackermann, M. Short-range interactions govern the dynamics and functions of microbial communities. *Nat Ecol Evol* **4**, 366-375, doi:10.1038/s41559-019-1080-2 (2020).

5 Dal Co, A., van Vliet, S. & Ackermann, M. Emergent microscale gradients give rise to metabolic cross-feeding and antibiotic tolerance in clonal bacterial populations. *Philos Trans R Soc Lond B Biol Sci* **374**, 20190080, doi:10.1098/rstb.2019.0080 (2019).

6 Park, J., Kerner, A., Burns, M. A. & Lin, X. N. Microdroplet-enabled highly parallel co-cultivation of microbial communities. *PLoS One* **6**, e17019, doi:10.1371/journal.pone.0017019 (2011).

7 Filkins, L. M. *et al.* Coculture of Staphylococcus aureus with Pseudomonas aeruginosa Drives S. aureus towards Fermentative Metabolism and Reduced Viability in a Cystic Fibrosis Model. *J Bacteriol* **197**, 2252-2264, doi:10.1128/JB.00059-15 (2015).

8 Fletcher, J. *et al.* The Citizen Phage Library: Rapid Isolation of Phages for the Treatment of Antibiotic Resistant Infections in the UK. *Microorganisms* **12**, doi:10.3390/microorganisms12020253 (2024).

9 Flynn, J. M., Phan, C. & Hunter, R. C. Genome-Wide Survey of Pseudomonas aeruginosa PA14 Reveals a Role for the Glyoxylate Pathway and Extracellular Proteases in the Utilization of Mucin. *Infect Immun* **85**, doi:10.1128/IAI.00182-17 (2017).
