# Supplementary material for: Harnessing droplet microfluidics and morphology-based deep learning for the label-free study of polymicrobial-phage interactions
Source: Commun Biol. 2025 Nov 12;8:1556. doi: 10.1038/s42003-025-08925-9 (PMC12612114; doi:10.1038/s42003-025-08925-9)
Supplement: Supplementary file 3 — Description of Additional Supplementary Files [file 42003_2025_8925_MOESM3_ESM.pdf]

Description of Additional Supplementary Files

File name- Supplementary Movie S1

File description – Example focus correction being applied before Z-stack imaging of every droplet with output of the python software displayed.

File name- Supplementary Movie S2

File description - Time-lapse brightfield images illustrating the growth of a co-culture within a droplet at different time points, with P. aeruginosa cells marked by green dots and S. aureus cells by red dots. Corresponding cell counts are plotted on the right-hand panel.

File name- Supplementary Movie S3

File description - Lysis of PA14 ΔflgK by P278 phage in droplet at MOI 0.6. Corresponding cell counts are plotted on the right-hand panel.

File name- Supplementary Movie S4

File description - Effect of P278 phage on polymicrobial populations of PA14 ΔflgK and MSSA476 at MOI 2.5. Corresponding cell counts are plotted on the right-hand panel.
